# Mechanisms of Fetal T Cell Tolerance and Immune Regulation

**DOI:** 10.3389/fimmu.2020.00588

**Published:** 2020-04-09

**Authors:** Elze Rackaityte, Joanna Halkias

**Affiliations:** ^1^Biomedical Sciences Graduate Program, University of California, San Francisco, San Francisco, CA, United States; ^2^Division of Neonatology, Department of Pediatrics, University of California, San Francisco, San Francisco, CA, United States; ^3^Eli and Edythe Broad Center of Regeneration Medicine and Stem Cell Research, UCSF, San Francisco, CA, United States

**Keywords:** fetal immunity, T cells, PLZF, Treg cell, inflammation, preterm birth, fetal inflammatory response

## Abstract

The developing human fetus generates both tolerogenic and protective immune responses in response to the unique requirements of gestation. Thus, a successful human pregnancy depends on a fine balance between two opposing immunological forces: the semi-allogeneic fetus learns to tolerate both self- and maternal- antigens and, in parallel, develops protective immunity in preparation for birth. This critical window of immune development bridges prenatal immune tolerance with the need for postnatal environmental protection, resulting in a vulnerable neonatal period with heightened risk of infection. The fetal immune system is highly specialized to mediate this transition and thus serves a different function from that of the adult. Adaptive immune memory is already evident in the fetal intestine. Fetal T cells with pro-inflammatory potential are born in a tolerogenic environment and are tightly controlled by both cell-intrinsic and -extrinsic mechanisms, suggesting that compartmentalization and specialization, rather than immaturity, define the fetal immune system. Dysregulation of fetal tolerance generates an inflammatory response with deleterious effects to the pregnancy. This review aims to discuss the recent advances in our understanding of the cellular and molecular composition of fetal adaptive immunity and the mechanisms that govern T cell development and function. We also discuss the tolerance promoting environment that impacts fetal immunity and the consequences of its breakdown. A greater understanding of fetal mechanisms of immune activation and regulation has the potential to uncover novel paradigms of immune balance which may be leveraged to develop therapies for transplantation, autoimmune disease, and birth-associated inflammatory pathologies.

## Introduction

A healthy human pregnancy, in which the fetus shares only half of its genes with the mother, is an impressive immunological feat. Non-inherited maternal antigens and a growing repertoire of self-antigens present a unique immune challenge to survival *in utero*. Suppression of responses to these antigens is critical to the maintenance of pregnancy (1–3), and the semi-allogeneic fetus relies on a specialized program of immune tolerance for survival *in utero*. Thus, humans have evolved redundant and dominant fetal mechanisms of tolerance that override our immune system's encoded ability to mount a protective response.

Human fetal development occurs within the anatomically distinct *in utero* environment defined primarily by the placenta, a chimeric organ composed of both fetal and maternal cells. Maternal immune adaptation to the semi-allogeneic pregnancy includes limitations on immune cell entry, activation, and function (4) as well as the appearance of uniquely tolerogenic cellular and molecular mechanisms [reviewed in (5)]. Features of pregnancy-induced immune tolerance are driven in part by the endocrine functions of the placenta as well as the state of physiologic hypoxia derived from the vascular anatomy of this organ. Finally, the placenta creates a protected niche which filters and limits fetal exposure to external antigens and microbes. Our understanding of placental biology has evolved from a barrier organ to one of feto-maternal communication [reviewed in (6)] and there is a growing appreciation for the role of the fetal immune system in the maintenance of a healthy pregnancy.

Murine models have contributed significantly to our understanding of maternal immune responses in pregnancy, however fetal immunity is poorly modeled in the mouse. Although thymus organogenesis is remarkably similar between the species, the functional output differs drastically during development, likely influenced by the relatively short murine gestation in comparison to that of humans. The first wave of murine T cells to exit the thymus are TCRγδ thymocytes destined for the skin around embryonic day 15 (7, 8). These cells are subsequently replaced by increasing thymopoeisis of conventional TCRαβ T cells which continue to populate the periphery until the end of the first week of life (9). In humans, TCRγδ and TCRαβ T cells, including regulatory T cells, exit the fetal thymus simultaneously and comparatively earlier than in mice [around 12–14 weeks of gestation; (10–12)]. Therefore, unlike mice, most T cell development in humans occurs *in utero*. Mice depend on a sustained thymic output of naïve T cells throughout their lifetime (13) and neonatal thymectomy results in severe impairment of immune responses to infection and autoimmunity (14–16). In contrast, humans primarily rely on expansion of existing naïve T cells post-natally, as incidental neonatal thymectomies during cardiac surgery do not give rise to autoimmune disease or an increased susceptibility to infection (13, 17, 18). Given these differences between mice and humans, this review will primarily focus on human adaptive immune development.

In humans, all cellular components of innate and adaptive immunity are present in the developing fetus. Adaptive immunity results from antigen-specific activation of T cells and B cells followed by the generation of long-lived memory cells capable of a robust recall response. Innate immunity, triggered by molecular pattern molecules, provides rapid protection from pathogens, and clears dying or damaged self-cells. Innate immune cells are also keystone initiators of the adaptive arm of immunity. T cells are classically activated by cognate peptide-MHC interaction with the T cell receptor (TCR), a process directed by professional antigen presenting cells (APCs) in the periphery. Fetal APCs, such as dendritic cells, can be activated in response to pathogen associated molecular pattern molecules (PAMPs), migrate between tissues and lymphatics, and robustly activate naïve T cells (19). These data indicate that fetal innate immune cells possess the capacity to direct T cell activation and differentiation *in utero*.

Fetal immunity serves specialized evolutionary goals adapted to the unique demands of gestation and thus differs significantly from that of the adult. Immune tolerance, while critical to survival *in utero*, must transition to a program of protective immunity in preparation for birth. The neonatal window bridges these two programs in remarkable concert, allowing for the establishment of tolerance to commensals, while also protecting the newborn from infection. In mice, this critical window of immune development is brief: tolerance to skin commensals for example, is preferentially established during the first week of life, coinciding with an abrupt influx of regulatory T cells [T_reg_; (20)]. In humans, this developmental transition may be longer and likely begins *in utero*, as maternal environmental exposures (e.g., to farming) result in a greater abundance of highly suppressive T_reg_ cells in neonatal umbilical cord blood (21), and childhood exposures protect from allergic disease in adult life (22–24). However, newborn protective immunity is not fully effective because neonates and infants exhibit a higher susceptibility to infection, the leading causes of mortality in these age groups (25).

**Figure 1:**
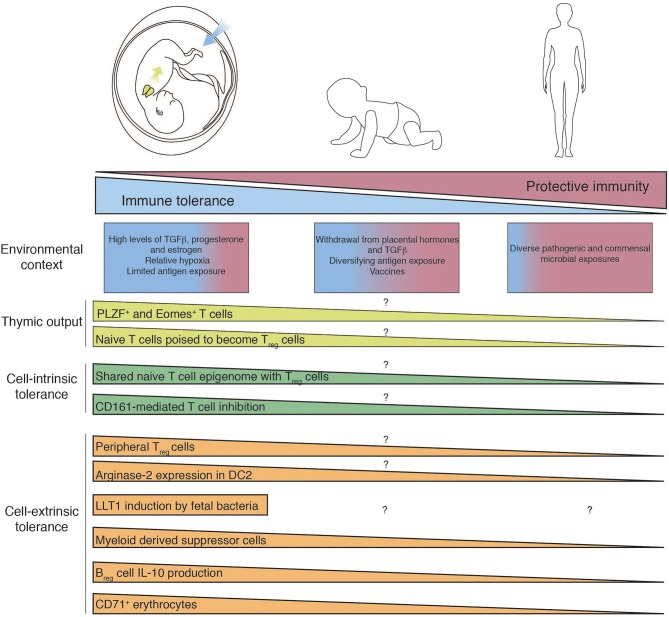
Mechanisms of fetal T cell tolerance and immune regulation. Recent thymic emigrants populate the periphery and encounter tissue-specific environments which differ between fetal, infant, and adult. T cell differentiation and function are governed by diverse cell-intrinsic and -extrinsic mechanisms of immune regulation, which are subsequently lost as *in utero* pressures for tolerance give way to the need for post-natal protective immunity. Question mark indicates features of immunity that have yet to determined.

This presumed failure of the infant immune system has been bolstered by studies of umbilical cord blood, in which the majority of T cells are naïve and fail to mount a pro-inflammatory response (26), and APCs exhibit an impaired ability to activate T cells (27). Umbilical cord blood, collected immediately after delivery, is composed of circulating fetal immune cells and is stereotypically distinct from the development of circulating neonatal immune cells in the first months of life (28). In contrast to immune cells in the blood, the infant intestine possesses a sizeable proportion of memory T cells (29–32) and lymphoid-derived fetal dendritic cells are capable of T cell activation (19). These recent reports of functional memory T cells capable of pro-inflammatory cytokine production in the fetal intestine indicate that the fetal immune system is not in essence immature, but is rather compartmentalized in its specialized function. Many of the unique features of the fetal immune system are lost with advancing age, for example the proclivity of naïve T cells to differentiate into T_reg_ cells (33), the presence of innate-like CD4 and CD8 T cells (29, 34, 35), and the thymus-derived capacity for T cell IL-8 production (36, 37). The aim of this review is to summarize the latest findings on the composition and function of fetal T cell immunity with a specific focus on mechanisms of tolerance and immune regulation (Figure 1). We discuss how the intra-uterine environment influences fetal immunity through unique adaptations and also examine experimental models to investigate these paradigms. Lastly, we consider the consequences of dysregulation of fetal immunity and how current findings could be leveraged to protect the fetus from perinatal inflammatory pathologies, such as preterm birth.

## Fetal T Cell Immunity

Thymic development begins by week eight of human gestation, and the first T cells begin to populate the periphery by 12–14 weeks of gestation (10, 38, 39). Unlike mice, both γδ and αβ T cells emigrate from the thymus simultaneously (7, 8, 38) and the appearance of human T_reg_ cells coincides with that of naïve T cells (11, 12, 16). Fetal T cell colonization in the periphery occurs in a state of relative lymphopenia in which naïve cells composed primarily of recent thymic emigrants begin to populate lymphoid and mucosal niches. Naïve T cells undergo rapid proliferation in response to homeostatic signals (40) similar to that seen in postnatal mice (41). While the vast majority of T cells in cord blood possess a naïve phenotype, healthy term cord blood contains memory T cells with adult-like inflammatory effector functions, albeit in very low proportion (42). Fetal adaptive immune memory was first reported in the fetal intestine (43–45), and memory T cells predominate in the infant and pediatric intestine (46), suggesting that early life adaptive memory is particularly abundant in mucosal tissues.

### Regulatory T Cells

Fetal immune tolerance is essential to the maintenance of pregnancy, achieved in large part by the ability of T_reg_ cells to suppress the activation, proliferation, and effector functions of a wide range of immune cells. T_reg_ cells [defined in humans by expression of FoxP3, CD25, and low or absent expression of CD127 (47, 48)] are strikingly abundant in peripheral lymphoid organs during the second trimester of human gestation, in stark contrast to neonatal and adult lymph nodes and adult peripheral blood cells (2, 12, 49, 50). Although thymic output of T_reg_ cells is similar *in utero* and after birth (33), fetal naïve T cells display an increased propensity to differentiate into T_reg_ cells upon antigen encounter in the periphery [induced T_reg_; iT_reg_; (33)]. Levels of TGFβ are higher in fetal than in adult lymph nodes, which potentiates the generation of iT_reg_ cells, and distinct fetal hematopoietic stem cells give rise to fetal T cells with the unique ability to differentiate into T_reg_ cells (51). A recent study demonstrated that fetal naïve T cells are already poised for tolerance and share a partial transcriptome and epigenome similar to that of T_reg_ cells, such as the heightened expression of the transcription factor *Helios* required for iT_reg_ function (52). These studies indicate that both cell-intrinsic and cell-extrinsic mechanisms promote the generation of a predominantly tolerant peripheral T cell response, essential for the maintenance of pregnancy.

Mold et al. demonstrated that fetal T cells rapidly proliferate to maternal and self-antigens in the absence of T_reg_ cells, indicating that fetal immune tolerance is an active process (33). The contribution of T_reg_ cells to perinatal immune tolerance is highlighted by IPEX (immune dysregulation, polyendocrinopathy, entheropathy, X-linked) syndrome, resulting from the loss of the T_reg_ lineage-defining transcription factor FOXP3. Onset of IPEX-related autoimmunity occurs in the second trimester, coinciding with the emergence of peripheral T cells and underscoring the importance of T_reg_- mediated tolerance to the survival of the fetus (53). That fetal T cells are actively suppressed by T_reg_ cells points to a broader capacity of fetal responses that are kept under tight control.

### T Helper Cell Type I (Th1) Cells

The need for active tolerance to self- and maternal antigens suggests that protective Th1 responses may be detrimental to a healthy pregnancy. Indeed, human cord blood from infants born preterm exhibits an enrichment of Th1 cells (54–56), thus implicating Th1 cells in the pathophysiology of the premature termination of pregnancy (1). However, a fetal immune response with Th1 polarization is also generated in response to maternal infections (57–60), indicating that protective immunity can be elicited under specific *in utero* conditions and is not always associated with a negative outcome.

Contrary to the predominantly naïve phenotype of T cells in cord blood, ~50% of CD4 T cells in the human fetal intestine exhibit a memory phenotype and robustly produce the Th1 cytokines IFNγ and TNFα (29–31). We recently reported that the majority of fetal effector memory T cells in the intestine express the transcription factor PLZF, are transcriptionally distinct from either conventional memory T cells or innate-like T cells, and are absent from the adult intestine (29). A greater proportion of PLZF^+^ CD4^+^ T cells produced Th1 cytokines as compared to conventional CD4 memory T cells, and this was most evident in the small intestine. Further, IFN-γ–producing PLZF^+^ CD4^+^ T cells were enriched in the cord blood of infants born preterm as well as in infants with gastroschisis, a congenital defect defined by chronic inflammation originating from the intestine. These data suggest that dysregulation of PLZF^+^ CD4^+^ T cells may contribute to pathologic systemic fetal immune activation.

While PLZF expression is sufficient to confer a memory phenotype and effector function in murine T cells (61, 62), its contribution to human T cell lineage commitment is not as clearly defined. Unlike classic innate-like T cells, functional maturation of fetal PLZF^+^ CD4^+^ T cells is not evident in the thymus and PLZF^+^ CD4^+^ T cells display a polyclonal TCR repertoire (29). Further, the capacity for activation in response to both TCR-dependent and cytokine-dependent signaling is also present in adult intestinal memory T cells (63). Thus, fetal PLZF^+^ CD4^+^ T cells share functional and transcriptional attributes with both conventional and innate-like T cells and may serve as a link between innate and adaptive immunity in the fetus.

A number of innate-like T cells capable of Th1 cytokine production are present in the human fetus, including γδ T cells, invariant natural killer T (iNKT) cells, and mucosa-associated invariant T (MAIT) cells. These cells share certain key features: a semi-invariant TCR repertoire, non-classical MHC restriction, and rapid production of inflammatory cytokines such as IFNγ and TNFα reviewed in (64). While iNKTs are enriched in mucosal tissues such as the intestine and the liver, they represent a small proportion of the total T cell compartment (<1%) in both fetal and adult tissues (65). Low proportions of Vα7.2^+^ CD161^+^ T cells with many attributes and characteristics consistent with MAIT cells were reported in the human fetus (<1% of CD3 cells) and were enriched in the fetal liver, small intestine, and lung (66). However, subsequent studies reported that a minority of fetal Vα7.2^+^ CD161^+^ T cells reacted with the riboflavin-bound MR1 tetramer (67). These findings indicate that broader specificity may exist in semi-invariant innate-like subsets in the fetus. Fetal semi-invariant γδ T cells are functionally pre-programmed to primarily produce IFNγ and have the capacity to generate Th1 responses to CMV (68). However, recent reports suggest that the repertoire of Vγ9Vδ2 T cells is distinct to the fetus and differs from that of the adult (69). In sum, a substantial number and diversity of conventional and innate-like fetal cells with the capacity for Th1 cytokine production are present in fetal tissues, suggesting a protective role at barrier sites during the critical neonatal window of development.

### T Helper Cell Type II (Th2) Cell

Historically, the perinatal immune system has been considered skewed toward Th2 responses and away from Th1 responses. However, much of the data supporting this notion was derived from murine studies. Immunization in neonatal mice favors Th2 memory pool formation (70, 71), even when immunized with a Th1 skewing adjuvant (72), and murine thymocytes exhibit an epigenetic predisposition for Th2 differentiation (73). Mirroring findings in neonatal mice, human naïve T cells derived from fetal cord blood and infant adenoids exhibited higher expression of GATA3 than that of adult T cells, and predominantly produced an unglycosylated isoform of IL-4 that is absent from adult Th2 cells (74). Similarly, the IL-13 locus of cord blood T cells is characterized by open chromatin and permissive epigenetic marks (75), suggesting a predisposition for Th2 differentiation. Human neonatal vaccine responses elicit predominantly Th2 type immunity reviewed in (76), similar to that seen in mice. However, Th1 and Th2 cytokine responses have been generated from cord blood naïve T cells *in vitro* (77) and neonatal vaccination with Bacillus Calmette-Guérin (BCG) produces an adult-like Th1 response (57, 58). Similarly, helminth and mycobacterial antigens induce an adult-like CD4 Th1 skewed response in cord blood of infants in regions endemic for schistosomiasis, filariasis, and tuberculosis (59). Placental malaria results in increased IFNγ- and TNFα-producing CD4 effector memory T cells that proliferate to malaria-specific antigens upon re-stimulation (60), indicating that Th1 immunity is possible in response to the appropriate stimuli. Th2 cell function and specificity has not been extensively investigated in the fetus outside of cord blood, though IL-4 producing cells are present in the fetal intestine (29, 30). In injury contexts, Th2 cells reduce inflammation and promote tissue regeneration [reviewed in (78)], which may be co-opted *in utero* to promote organogenesis. Thus, while some neonatal vaccine responses induce a Th2 bias, a Th1 response can be elicited under specific conditions, broadening our prevailing understanding of human fetal adaptive immunity.

### T Helper Cell Type 17 (Th17) Cells

The role of Th17 cells has not been extensively studied in the context of human fetal development. Term infant cord blood is uniquely enriched in a population of CD161^+^ CD4 T cells which preferentially gives rise to Th17 cells under *in vitro* differentiation conditions (79). Further, cord blood naïve CD4 T cells exhibit an enhanced capacity for Th17 differentiation compared to adult cells (80). However, term cord blood CD4 T cells are not capable of either IL-17A or IL-17F production despite the presence of CCR6^+^ effector memory cells with high levels of *RORC* expression (42). In contrast to circulating fetal immune cells, IL-17 production is restricted to CD4^+^ T cells expressing the transcription factor PLZF in the mesenteric lymph node and to a lesser extent in the small intestine, supporting the spatial segregation of fetal effector function (29). Although IL-17 can be produced by human immune cell subsets other than T cells (81), IL-17 production was not appreciated in fetal γδTCR T cells (82) and the capacity of human fetal memory CD8 T cells to produce IL-17 has not yet been reported.

### CD8 T Cells

Cord blood naïve CD8 T cells are transcriptionally and epigenetically distinct from that of the adult with a proclivity for proliferation and innate immune responses. These cells express neutrophil-associated and antimicrobial peptide genes, but exhibit a decreased cytotoxic response in comparison to adult cells (83). Fetal CD8 T cells that express KIR/NKG2A and Eomes possess innate-like properties and produce IFNγ in response to IL-12 and IL-18 (34, 35). In parallel, the fetus is also capable of conventional CD8 T cell responses comparable to those of the adult in response to specific maternal infections. Fetal CD8 T cells mount an antiviral-specific response to hepatitis B (84), hepatitis C (85), HIV (86–88), and cytomegalovirus infection, and effectively differentiate into memory cells capable of perforin-production (89). Placental malaria also induces antigen-specific memory CD8 T cell proliferation, though this does not result in increased inflammatory cytokine production (60). Antigen-specific CD8 T cell responses in the fetus and neonate indicate that early-life immunization is possible against specific pathogens. The fetal cytokine environment may limit the activation of CD8 T cells, as TGFβ has been shown to suppress cytotoxic CD8 T cell differentiation induced by IL-15 (90). Fetal CD8 T cells may be highly atypical, as they possess the capacity to produce IL-4 when stimulated in the absence of IL-2 and IL-12, a feat reminiscent of iNKT cells that is absent from adult CD8 T cells (91). In mice, neonatal CD8 T cells originate from distinct hematopoietic stem cell sources than adult CD8 T cells and exhibit rapid and short-lived responses as compared to adults (92, 93), exhibiting a layering of immune function similar to that of human fetal CD4 T cells (51). Together, this evidence points to fetal-specific features for innate-like CD8 T cells which likely play a role in perinatal immune protection.

## Fetal Mechanisms of T Cell Regulation

The remarkable potential of fetal T cells to generate inflammatory responses suggests that strong and redundant mechanisms must exist to preserve a tolerogenic environment required for a successful pregnancy. Indeed, the fetal immune system has adapted and repurposed unique cell-intrinsic and cell-extrinsic means to limit inflammation (Figure 1).

### Cell-Intrinsic Mechanisms

Fetal naïve T cells have a greater capacity to differentiate into T_reg_ cells, which may in part be explained by a higher sensitivity to TGFβ indicated by high levels of SMAD2/SMAD3 phosphorylation in the unstimulated state (49). Lin28b, a repressor of let-7 microRNAs that target TGFβ signaling mediators, is highly expressed in fetal naïve T cells and is required for the increased propensity toward Treg cell differentiation (49). Unlike adult naïve T cells, fetal naïve T cells have an increased propensity to differentiate into T_reg_ cells even in the absence of high levels of exogenous TGFβ (49), suggestive of additional cell intrinsic mechanisms such as a poised epigenome. The predisposition of fetal naïve cells to differentiate into T_reg_ cells is driven by higher expression of and chromatin accessibility at the Helios (IKZF2) locus, and ablation of Helios in fetal naïve T cells results in poor T_reg_ differentiation and impaired function (52). These studies point to additional cell-intrinsic differences in fetal naïve T cells that contribute to a predominant program of immune tolerance and regulation in response to antigen encounter *in utero*.

T cell activation is governed by productive and simultaneous TCR- and co-stimulatory molecule signaling. Molecules that inhibit T cell activation (co-inhibitory) are expressed either in conjunction with co-stimulatory molecules or following successful activation reviewed in (94). Fetal intestinal memory PLZF^+^ T cells express high levels of the surface C-type lectin receptor CD161 (29). In adults, engagement of CD161 inhibits human NK cells (95–97), but exerts co-stimulatory effects on iNKT cells (98), MAIT cells (99), and displays no consistent influence on adult polyclonal T cell activation (95, 100). It is thus striking that ligation of CD161 by two different monoclonal antibodies inhibited IFNγ production in response to TCR activation in fetal PLZF^+^ T cells (29). These reports point to a unique repurposing of the CD161 axis in the fetal context, the signaling mechanisms of which remain to be explored.

Despite strong inhibition following TCR-dependent stimulation, CD161 ligation has no effect on cytokine-mediated T cell activation, which remains a critical area of study. The differential enrichment of numerous negative T cell regulators (*DUSP4, DUSP5, DUSP6, LRIG1*, and *DTX1*) in the gene signature of fetal intestinal PLZF^+^ CD4^+^ memory T cells (29) suggests the existence of additional cell-intrinsic mechanisms of regulation to promote immune homeostasis *in utero*. In particular, the transcriptome of fetal PLZF^+^ CD4^+^ T cells was differentially enriched for multiple surface molecules with known inhibitory functions such as PD1 (29). Thus, determining the extent to which known and novel co-inhibitory molecules can limit fetal T cell activity is a promising therapeutic avenue for birth-related pathologies, as many inhibitors to these receptor-ligand interactions are already in use and continue to be developed for cancer immunotherapy.

### Cell-Extrinsic Mechanisms

T cell immunity is regulated in large part by the surrounding environment, such as the antigen presenting cells which directly engage with and efficiently activate T cells. Given their central role in T cell activation and differentiation, it is thus not surprising that they also contribute to fetal immune regulation. For example, type II fetal dendritic cells (fDC2) limit the ability of T cells to produce IFNγ, IL-2, IL-17, TNFα, and other inflammatory cytokines (19). Inhibition of TNFα production by fetal T cells is achieved through upregulation of Arginase-2 on fDC2s (19), however the mechanisms of inhibition of other inflammatory cytokines have yet to be determined. Production of IFNγ by fetal T cells may be limited by intestinal macrophages expressing high levels of LLT1, the natural ligand of CD161 (29). Exposure to fetal-specific *M. luteus* induces LLT1 on fetal antigen presenting cells (101), suggesting that commensal fetal bacteria may modulate this axis to promote intestinal immune tolerance. Further, suppressive functions of intestinal macrophages are well-described in adults (102–104) and it is likely that similar mechanisms are also employed by the fetal immune system.

Additional human myeloid cell populations contribute to T cell tolerance *in utero*. Myeloid-derived suppressor cells (MDSCs) are a heterogeneous subset of monocytic and granulocytic/neutrophilic cells that limit T cell and NK cell immunity in tumors and during the course of infection reviewed in (105). Neutrophilic MDSCs capable of suppressing Th1, Th2, and Th17 responses are enriched in cord blood and rapidly decrease to adult levels during infancy (106, 107), with levels dropping drastically after the first month of life (108). These MDSCs are capable of phagocytosis of *E. coli*, yet produce high levels of TGFβ and strongly suppress T cell proliferation (109), a process which requires cell-cell contact (108). Further, BCL-2 mediates lower rates of apoptosis among cord blood MDSCs, suggesting that their prolonged survival may promote a tolerizing environment even after the resolution of bacterial infection (109). It is plausible that C-type lectins may also be involved in regulating fetal MDSCs, because anti-inflammatory IL-10 production in these cells is regulated by co-triggering TLR-MyD88 and C-type lectin receptor-Syk-dependent pathways (110). Neutrophils migrate to tissues at steady-state and support organ function (111), yet their role in fetal development has not been investigated.

The lymphocyte compartment contributes to the modulation of fetal T cells through the activity of T_reg_ cells and regulatory B (B_reg_) cells. Fetal tolerance is maintained in large part by active T_reg_ cell-mediated suppression of T cell activation (33), yet whether fetal T_reg_ cells exhibit classic or unique suppressive mechanisms has not been formally demonstrated. Further, while T_reg_ cell specificity for non-inherited maternal antigens has been proposed, the question of antigen-specific suppression requires further study. B_reg_ cells [reviewed in (112)] are enriched in the fetus and an immunosuppressive role for B_reg_ cells has been proposed in the context of maternal tolerance (113, 114). In neonatal mice, IL-10-producing B_reg_ cells limit the ability of dendritic cells to produce inflammatory cytokines, which can be induced by broad TLR stimulation of dendritic cells and is paradoxically induced by type I interferon conditioning of B cells in acute inflammatory contexts (115, 116). In humans, B_reg_ cells are highly abundant in cord blood, produce IL-10, and are capable of suppressing CD4 T cell responses in an IL-10-dependent manner through CTLA4 and CD40 mechanisms (117). Cord blood B_reg_ cells exhibit higher IgM expression as compared to adult blood and can inhibit both Th1 and Th2 responses (118), however IgM-expressing B cells are more prevalent in infant than in fetal intestines (32). Thus, fetal lymphocytes contribute to the maintenance of tolerance in pregnancy through the generation of T_reg_ and B_reg_ cells.

Human and murine neonatal peripheral blood cells exhibit high proportions of transferrin receptor expressing (CD71^+^) erythrocytes with the unique ability to suppress T cells (119). Similar to fetal dendritic cells (19), CD71^+^ erythrocytes mediate suppression through Arginase-2, suggesting that arginine depletion is a key mechanism of immune regulation in the fetal environment (119). Although the CD71 axis has not been explored in human fetal tissues, depletion of CD71^+^ erythrocytes results in myeloid cell activation and increased production of TNFα in the intestine of neonatal mice. Because activation of intestinal myeloid cells in response to CD71^+^ cell depletion does not occur in germ-free or antibiotic treated neonatal mice, this suggests that CD71^+^ cells may contribute to tolerance to commensal colonization, a critical immune adaptation in early life (119). Further, a subset of CD71^+^ erythrocytes which express high levels TGFβ may contribute to the increased conversion of naïve T cells into T_reg_ cells. Ablation of T_reg_ cells results in higher proportion of CD71^+^ erythrocytes and these erythrocytes compensate by increasing expression of TGFβ after transient CD71^+^ cell depletion (120). The interdependence of neonatal T_reg_ cells and CD71^+^ erythrocytes points to a repurposing of conserved axes that limit T cell immunity.

## *in utero* Environment Contributes to the Regulation of Fetal T Cells

The highly controlled biological environment *in utero* permits the development of the fetal immune system in a state of active immune suppression, while also promoting fetal-specific mechanisms of immune protection. For example, physiologic intra-uterine hypoxia could contribute to fetal immune regulation by limiting the ability of myeloid cells to activate T cells [reviewed in (121)]. Tissue hypoxia also enhances T_reg_ numbers and function (122), pointing to multiple mechanisms by which fetal hypoxia contributes to immune tolerance *in utero*.

The developing human fetus is exposed to increasing levels of placental hormones with potent immunomodulatory effects throughout gestation. The placenta produces the steroid hormones estrogen [predominantly estradiol (E2) and estriol (E3)] and progesterone (P4), which are critical to maternal tolerance of the fetus (123–131). Despite high levels of placental hormones in the fetal circulation, little is known about their effect on fetal T cells. P4 promotes differentiation of naïve cord blood T cells into T_reg_ cells and suppresses the differentiation of Th17, yet has little effect on adult T cells, suggesting that the sensitivity to P4 is lost in adulthood (125). A similar immunosuppressive role has been described for E2 in mice (123, 130, 132), yet the direct effect of E2 on human fetal T cells remains to be explored.

Recent reports suggest that the repertoire of antigens seen by the fetus is broader than previously appreciated. Fetal T cell responses to self- and maternal-antigens are more robustly skewed toward the T_reg_ cell lineage compared to adults (33), yet observations of inflammatory recall responses in the fetus suggest a more varied antigen exposure *in utero*. Bacteria have been detected in amniotic fluid and the placenta through culture-independent studies of these tissues (133–136), though this remains a controversial area of research as other groups were unable to replicate these findings (137, 138). We recently reported that bacteria are variably present in the fetal intestine through high resolution scanning electron microscopy, sequencing, and viable isolation methods. The presence of *Micrococcus luteus* in fetal meconium was correlated with a higher proportion of intestinal PLZF^+^ CD161^+^ memory T cells and limited their ability to produce IFNγ as compared to non-fetal *M. luteus* species (101). This finding is supported by a study that demonstrated the requirement of a microbiome for the development of PLZF^+^ T cells in mice (139). Thus, maternal and fetal commensal bacteria could potentially play a role in modulating fetal immunity and limiting *in utero* inflammation. In addition to bacteria, the fetus is exposed to allergens in amniotic fluid (140), and cord blood T cells exhibit recall responses to ovalbumin, cockroach, mouse, house dust mite, and *Fel d* cat allergen (140–144). These studies underscore the ability of fetal T cells to recognize a broad range of antigens and that their responses can be regulated by highly adapted fetal bacteria. In addition to fetal-specific, cell-intrinsic mechanisms of regulation, the development of human T cell immunity is strongly shaped and influenced by a regulated set of exposures within the *in utero* environment.

## Consequences of Fetal Immune Dysregulation

Despite dominant and redundant mechanisms that control T cell immunity *in utero*, fetal T cell activation can occur in pathologic contexts and contribute to the initiation of the fetal inflammatory response. Preterm birth (PTB), defined as birth before 37 weeks of gestation, affects 1 of every 9 newborns, and is the leading cause of childhood death under the age of 5 worldwide (25, 145). Preterm labor (PTL), the main cause of PTB, is more frequent when a fetal inflammatory response is elicited (146, 147), and most of the complications associated with death and disability in preterm infants are triggered by inflammation (56, 148–151). Despite the impact of infection and inflammation on the survival and long-term outcome of preterm infants, little is known about the mechanisms that drive fetal immune responses and their contribution to perinatal immune dysregulation.

Increased proportions of activated T cells and reduced suppressive T_reg_ cell activity are evident during fetal inflammation (54–56, 152) and maternal-reactive fetal memory Th1 cells are implicated in the pathophysiology of PTB (1). Activated CD4 and CD8 T cells derived from the cord blood of preterm infants induce uterine cell line contractility *in vitro*, an effect which can be rescued by anti-TNFα or anti-IFNγ antibodies (1). Thus, T cell activation and specifically Th1 cytokines may drive the fetal inflammatory response associated with PTB. Animal models of inflammatory PTB identify the fetal intestine as a site of immune activation (153–156), indicating that mucosal T cells in contact with amniotic fluid are ideally situated to contribute to the fetal inflammatory response. Indeed, fetal PLZF^+^ CD4 T cells, which specifically accumulate in the fetal intestine, are enriched in the cord blood of preterm infants and exhibit a heightened capacity for both TNFα and IFNγ production as compared to term infants (29). A similar pattern of PLZF T cell accumulation is evident in the CB of infants with gastroschisis, a congenital abdominal wall defect characterized by intestinal T cell activation (29). Taken together, these findings point to a role for PLZF^+^ CD4 T cells as mediators of the fetal inflammatory response.

Pathology associated with activation of fetal intestinal T cells is exemplified in necrotizing enterocolitis (NEC), a co-morbidity of PTB. NEC infants exhibit higher proportions of effector memory CD4 T cells in the lamina propria of the intestine and these cells have a heightened capacity to produce TNFα and reduced capacity for IL-10 production (30). While moderate TNFα levels promote intestinal epithelial maturation, dysregulation of intestinal T cells leads to tissue-damaging effects of TNFα (30). A breakdown in cell-intrinsic mechanisms of human fetal tolerance has not been investigated in this context, but lack of sufficient T_reg_ populations may contribute to disease development in NEC (157). Murine models of NEC demonstrate that CD4 T cell activation is critical to the initiation of inflammation (158). Transfer of NEC intestinal lymphocytes was sufficient to induce Th17 inflammation in otherwise naïve mice. Decreased T_reg_ cell levels were observed and could be rescued by retinoic acid supplementation (158), suggesting that loss of tolerance is a key tenet in this pathology. Ample evidence supports a role for dysregulation of T cell immunity in perinatal pathologies, therefore understanding the mechanisms that keep T cells in check may uncover novel therapeutics for these diseases.

## Conclusion

The developing human immune system is not forged in an antigenic vacuum, but rather educated in the presence of self-, maternal-, and environmental antigens. It is therefore not surprising that protective adaptive immunity critical to perinatal surveillance and defense is generated concurrently with a program of immune tolerance *in utero*. The ability of fetal T cells to mount a protective and, in pathogenic contexts, dangerous inflammatory response reinforces the existence of fetal-specific adaptations of immune regulation. Cell intrinsic mechanisms of control are further bolstered by tolerizing pregnancy factors such as the hormonal milieu, hypoxia, and relative depravation of environmental antigens. Insight into these tolerizing mechanisms and their broader applicability to limit human immunity will likely have a major impact on human health.

Early-life immune exposures and responses strongly shape lifelong health, thus increasing the urgency to understand the development of human fetal immunity. There is a growing need to develop methods to study human samples and for innovative model systems to explore the mechanisms of disease. Discoveries in human fetal immunology for the past decades have primarily come from studies in human cord blood, and will continue to serve as an important investigative tool for biomarker and therapeutic development. The study of perinatal inflammatory pathologies relies primarily on large animal models (e.g., sheep, macaques) that possess a full adaptive immune response similar to that of the developing human to explore mechanisms of injury. While cord blood will continue to inform predictive models for disease development, a global understanding of fetal immune development and its progression into childhood and beyond is still lacking. The study of human fetal tissue has served a critical, non-redundant role in our understanding of human *in utero* development, leading to groundbreaking discoveries in fetal immunity and organogenesis (19, 29–33, 51, 52, 101). When obtained through ethics-board approved protocols and informed consent, donated human fetal tissue offers a wealth of information which has yet to be fully interrogated. Continuing advances in single cell phenotyping as well as cell and tissue culture will accelerate investigation of human development and the translational impact of these discoveries.

Both protective and tolerogenic human T cell immunity begins *in utero* and the opportunity to intervene in immune developmental trajectories before the onset of disease underscores the importance of studying fetal T cell behavior and regulation.

## Author Contributions

ER and JH researched and wrote the manuscript.

### Conflict of Interest

The authors declare that the research was conducted in the absence of any commercial or financial relationships that could be construed as a potential conflict of interest.
